# Short-Term Adaptations to Lifting and Gait Kinematics When Using a Passive Back-Support Exoskeleton

**DOI:** 10.1007/s10439-025-03770-7

**Published:** 2025-06-18

**Authors:** Duleepa Subasinghe, Jessica Aviles, Amir Mehdi Shayan, Divya Srinivasan

**Affiliations:** 1 Department of Industrial Engineering, Clemson University, Freeman Hall, Clemson, SC 29634 USA; 2 Department of Bioengineering, Clemson University, Clemson, SC 29634 USA

**Keywords:** Trunk kinematics, Lifting strategies, Postural stability, Gait control

## Abstract

Prior work quantifying the biomechanical effects of back-support exoskeleton use has mostly focused on the effects of brief periods of exposures to exoskeletons. Hence, there is currently limited understanding of how movement kinematics may be altered by more prolonged exposures. We assessed the effects of a 75-min exposure to a passive back-support exoskeleton on adaptations to lifting strategies, gait kinematics, and postural stability. Twelve participants performed tasks in an ABA protocol—measurements were obtained before (Pre-EXO phase), during (EXO-adaptation phase), and after exoskeleton-use (Post-EXO phase). A piecewise linear regression model was used to estimate changes to the dependent variables within and between each phase. Trunk range of motion (ROM), peak trunk flexion angle, and flexion velocity showed significant decrease (6–8%) on introduction of the exoskeleton, and significant reversals on doffing the exoskeleton. However, there were no significant adaptation effects (changes during EXO-adaptation phase) to trunk kinematics. For gait, a more cautious gait pattern was observed during exoskeleton-use: step length decreased, step width increased, minimum toe clearance increased, and hip ROM decreased, compared to the baseline Pre-EXO phase. These measures also reversed on doffing the exoskeleton and demonstrated further carry-over effects during the Post-EXO phase. However, no significant adaptations were evident in gait kinematics. Exoskeleton introduction, use, and doffing did not alter the cycle-to-cycle variability of trunk kinematics, or postural stability during static stance and maximum leans. These findings can help guide the practical development of training and use protocols for safe exoskeleton use in occupational settings.

## Introduction

Back-support exoskeletons are wearable systems that provide structural support and use external torques to assist trunk extension. These exoskeletons aim to alleviate physical demands during tasks such as repetitive lifting, by reducing the muscular activity by 6–48% [1–5]. Passive exoskeletons achieve this using mechanical components like springs and dampers, or elastic elements, or flexible beams, whereas active exoskeletons use powered actuators [6]. Irrespective of actuation type, back-support exoskeletons have been shown to be effective in decreasing trunk extensor muscle activity by 9–28% [7–10], spinal compression forces by 8–17% [11–14], and metabolic cost by 6–17% during repetitive lifting [7, 15, 16]. However, while exoskeletons have been shown to be promising in terms of reducing trunk extensor muscle activity, whether there are other additional factors that may affect how exoskeleton-use can impact long-term low-back injury risk are beginning to be explored. For example, studies have explored whether the external assistance applied through either torque generators or elastic elements of back-support exoskeletons may cause different kinematic strategies for task completion (e.g., altered trunk-pelvis coordination [17, 18]); whether exoskeletons cause redistribution of loads among the low-back musculature (e.g., altered neuromuscular coactivations [19]); as well as whether exoskeleton-use alters spinal stability (e.g., changes in short- and long-term maximum Lyapunov exponents [18, 20, 21]).

In terms of kinematics, while some studies have reported that trunk kinematics (peak flexion) is minimally affected by the use of back-support exoskeletons (e.g., [16, 22, 23]), others have reported a decrease in peak flexion angle, angular velocity, and trunk range of motion (RoM) during exoskeleton use (e.g., [12, 20, 24, 25]), and alterations to preferred lifting strategy (e.g. [8, 15, 26]). Studies have also reported increased cycle-to-cycle variability in the thoracic spine kinematics when using an exoskeleton for bending/lifting [27].

Furthermore, researchers have also explored how back-support exoskeletons may unintentionally affect other aspects of movements beyond trunk flexion-extension, such as gait and postural stability, since application of external torques and/or decreased joint mobility at the hips may also influence balance and gait control strategies, thereby potentially disrupting gait patterns and/or modifying fall risk [28–31]. Correspondingly, studies on whether back-support exoskeletons affect gait and postural stability have reported changes in specific balance and gait measures, indicating deterioration in gait and balance control when using exoskeletons. Park et al. [30] reported increased center-of-pressure (COP) median frequency and mean velocity during bipedal stance and reduced COP displacement and sway area during unipedal stance. In terms of differences in spatiotemporal parameters of gait between exoskeleton and control conditions, Park et al. [29] reported decreased step length, increased step width, increased stride time and less time spent in swing phase within each stride; while Baltrusch et al. [15, 31] reported decreased stride length, with the use of back-support exoskeletons. Changes in lower-limb joint kinematics and kinetics have also been reported with back-support exoskeleton use [28]. However, most prior work on objective quantification of biomechanical outcomes with exoskeleton use has focused on the effects of brief periods of exposures to exoskeletons. There is currently limited understanding of how any positive/negative effects of exoskeleton-use on movement patterns may be altered by more prolonged exposures, and the implications of such adaptations to performance and safety.

The extent of user adaptations to prolonged use of back-support exoskeletons in industrially relevant tasks, such as in manual material handling, remain unknown. Most previous laboratory studies on back-support exoskeletons have included familiarization periods during which users are exposed to and trained on exoskeleton-use, prior to data collection. While the purpose of such familiarization periods may have been to achieve steady state behaviors prior to data collection, there is no basis or consensus in the literature for how long users need, to adapt to exoskeleton-use, before beginning to exhibit steady-state task strategies. As a result, exoskeleton familiarization periods have ranged from 5–7 min [23], 10 min [12] to 15–30 min [16], or have been unspecified [6, 9, 10, 32]. Among field studies of back-support exoskeleton use, a very recent study by Jakobsen and colleagues [33] report on a 5-week graded training protocol, that increased workers’ exposure to the exoskeleton from 7.5 h/week in week 1 to 37 h/week by week 5. Comparing biomechanical outcomes from week 1 vs. week 5 of their participants, the authors reported significantly lower peak trunk extensor muscle activity and altered kinematic strategies (stoop vs. squat, depending on magnitude of loads handled).

The initial familiarization and training play an important role in participants’ acceptance of the exoskeleton and should include technology demystification, techniques, potential, limits, donning/adjusting/doffing, and training scenarios, which have been highlighted by Moyon and colleagues [34]. Moyon and colleagues explored familiarization times for an arm-support exoskeleton by allowing 11 participants to individually choose their optimal familiarization times in field settings. However, to what extent their study of familiarization to an arm-support device in field settings would transfer to other tasks and different exoskeleton types is currently unknown. A recent review of training and familiarization protocols for exoskeletons comments on the lack of standardization of familiarization periods and protocols, and the inconsistencies in the various approaches that have been applied in prior work [35]. Such variability in familiarization periods makes it difficult to determine whether reported biomechanical (or subjective) measures across different studies and different exoskeleton designs are consistent, stable, and comparable outcomes. As a result, whether reported outcomes from short-duration lab studies are valid representations of acquired longer-term behavioral patterns from exoskeleton users in the field has also been questioned. Moreover, while most of these studies utilized various familiarization periods and protocols prior to conducting their research investigations, almost none of them were focused on measuring objective biomechanical outcomes during the familiarization periods, to investigate how user-behaviors were changing, and whether they had reached steady-state. Consequently, a stronger scientific basis is also needed to standardize familiarization protocols and periods for industrial adoption of exoskeletons, to ensure optimal performance and safety.

Previous research explicitly exploring the effects of familiarization time on stabilization of biomechanical outcomes has largely been conducted for hip and ankle exoskeletons and reported that stabilization of metabolic energy cost is associated with the exoskeleton type, familiarization task, and familiarization period [36–38]. Specifically, Panizzolo et al. demonstrated that metabolic cost changes can stabilize when using a hip exoskeleton within three 20-min familiarization walking sessions, while Galle et al. found that the stabilization time for metabolic energy expenditure when wearing an ankle exoskeleton was 18.5 min. Contrary to these, a prior investigation of a whole-body powered exoskeleton has shown that novice movement patterns did not match that of experts even after 3 sessions of exoskeleton use [39]. Finally, recent work has shown that in addition to training time (~ 109 min, as reported by this study), major metabolic and biomechanical benefits of exoskeleton use were also made possible by customizing the training to individual users [38]. While these studies have explored the effects of familiarization time on stabilization of a variety of biomechanical outcomes during gait, to our knowledge, no study has systematically explored the impact of familiarization time on movement kinematics involving occupational back-support exoskeletons used for manual material handling purposes.

Hence, the purpose of this study was to assess the effects of a prolonged (75 min) exposure to a passive back-support exoskeleton on movement kinematics during lifting, gait, and postural stability. ‘*Adaptation*’ in this study was defined as changes/adjustments observed in movement kinematics as individuals experienced use of an exoskeleton, and operationalized as the relative change in their movements over the duration of the exoskeleton exposure, compared to the start of the exposure. Thus, an individual’s initial reaction to donning the exoskeleton (difference from no-exoskeleton to exoskeleton phases), adaptation over the duration of the prolonged exposure (change over time during exoskeleton exposure), as well as the effects of doffing the exoskeleton and returning to a no-exoskeleton condition (difference from exoskeleton to no-exoskeleton phases), were assessed using an ABA protocol by quantifying changes in trunk and lower-limb kinematics over the course of the experiment. A 75-min exposure period was conservatively chosen, based on the available literature that demonstrated effective adaptation to hip and ankle exoskeletons within 60 min. We expected that peak trunk flexion and trunk velocity would initially decrease with exoskeleton donning, that they would continue to change over the exposure period, and that they would return to pre-exoskeleton exposure levels once the exoskeleton was doffed. We also expected hip flexion to decrease during gait; and postural stability and gait spatiotemporal parameters to be altered with exoskeleton donning, but that these changes would adapt to pre-exposure levels with prolonged exposure.

## Methods

### Participants

A convenience sample of twelve healthy young adults (6 M, 6F) were recruited from the university and local community in Greenville, SC, to participate in this study (participant demographics reported in Table 1). Inclusion criteria required participants to have no prior experience with using exoskeletons, no self-reported musculoskeletal disorders or injuries in the past 12 months, and not have undergone back, shoulder, hip, or knee surgeries. This study was approved by the Clemson University Institutional Review Board, and all participants signed informed consent prior to participation.

**Table 1:** Participant characteristics are reported as group means (standard deviation)

	Males (*n* = 6)	Females (*n* = 6)	*p*-value of gender difference
Age (years)	24.8 (3.4)	28 (6.8)	N/A
Stature (cm)	173.0 (6.3)	154.4 (7.8)	0.001
Body mass (kg)	81.5 (4.8)	57.2 (11.0)	< 0.001

### Exoskeleton

The exoskeleton used in this study was a passive back support soft exosuit (Apex, HeroWear, Nashville, TN, USA), designed to provide assistance during lifting tasks. This device is a commercially available back-support exoskeleton that is beginning to be widely used in industrial settings. The Apex exoskeleton weighs 1.5 kg and consists of elastic bands that run along the back, connecting straps on the upper body section to sleeves on the thighs (Fig. 6). The band sizes can be chosen according to participant anthropometry, and device assistance levels (low, medium, high) can be selected based on user preference, to individualize the exoskeleton to the user. In this study, while device sizing was configured according to user-anthropometry by following manufacturer recommendations, we standardized the assistance level to be ‘medium’ across all participants. This was done to control any effect of assistance level on the rate of adaptation.

### Study Overview and Protocol

**Fig. 1:**
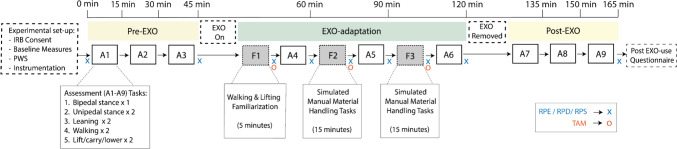
Flowchart of the study design. Assessments are indicated as A1–A9 and exoskeleton familiarization periods as F1–F3 in the order of they were carried out. The set of five tasks defined under the Assessment 1 (A1) was performed by participants in all of the 9 assessments listed. Bipedal task was performed in A1, A4, and A7. The timeline is approximate and reflects the average time spent in each phase. The ‘x’s and ‘o’s represent *RPE* rating of perceived exertion, *RPD* rating of perceived discomfort, *RPS* rating of perceived balance, *TAM* technology acceptance model

A repeated-measures study was designed, consisting of a single 4-h session, in which participants received exposure to the Apex exoskeleton (EXO) in an ABA protocol (Fig. 1). They first completed a set of tasks without using an EXO (A: *Pre-EXO* phase), followed by a series of familiarization periods and intermittent assessments conducted when they used an EXO (B: *EXO*-*adaptation* phase), and finally completed a set of tasks after doffing the EXO (A: *Post-EXO* phase). The tasks in both the familiarization periods and assessments were chosen to reflect possible activities and task demands regularly encountered in manual material handling, such as lifting and carrying. After signing the informed consent form, participants completed basic demographic and physical activity questionnaires, as well as anthropometric and strength assessments. They were then instrumented with a custom full-body motion capture marker set. Following this, we obtained participants’ preferred walking speed (PWS) according to the protocol described in Wu et al. [40] prior to the commencement of the experimental data collection.

During the experimental data collection, participants completed a total of 9 assessments: Assessments 1–3 (A1–A3) and Assessments 7-9 (A7-A9) were conducted when they were not wearing an exoskeleton, in the *Pre-EXO* and *Post-EXO* phases, respectively. Assessments A4–A6 were conducted while the participants were wearing the Apex exoskeleton, to measure the effects of time (adaptation) on user responses (*EXO-adaptation* phase). All assessments were identical, and included balance, gait, and repetitive lifting/lowering trials. During the EXO-adaptation phase, i.e., in between A4–A6, participants were exposed to three exoskeleton familiarization periods (F1–F3). The first familiarization period (F1) occurred immediately after the participant donned the exoskeleton for the first time, before the first EXO assessment (A4). During F1, participants performed a general (unstructured) 5-min familiarization task, where they lifted and carried a box with a weight equal to 10% of the participant’s body mass, back and forth on a level walkway. This familiarization period was meant to replicate procedures followed in other laboratory studies that provided a short familiarization period prior to assessing EXO performance effects [41]. The second and third familiarization periods (F2, F3) were designed to follow A4 and A5 respectively, and consisted of 15 min of a simulated material handling task. We collected subjective measures, including Ratings of Perceived Exertion (RPE) [42], Ratings of Perceived Discomfort (RPD) [43] (for shoulder, upper back, lower back, and legs), Ratings of Perceived Stability (RPS) [44], and Technology Acceptance Model (TAM) [45] based on exoskeleton acceptance, at various time points throughout the session. These measures, along with additional questions on perceived performance, range of motion, and stability, were recorded to capture participants’ responses at key stages of the protocol. For approximate timings of each assessment, please refer to Fig. 1.

### Experimental Task Setup

Each Assessment consisted of three balance tasks, gait task, and a lifting/lowering task, which were performed in the same order for each assessment.

Balance: The three balance tasks included quiet bipedal standing, quiet unipedal standing task, and a maximum voluntary leaning task. During the bipedal quiet standing, participants were asked to stand on an embedded force plate in an upright position, with both feet closer together, arms abducted ~ 60°, and looking straight ahead for 30 s. Each quiet unipedal stance was 20 s and was performed with participants’ preferred balance foot with their non-standing leg slightly flexed, and arms abducted ~ 60°.During maximum leaning trials, participants were asked to stand with their feet close together, arms crossed over their chest, and facing straight forward. Then they were asked to lean as far as possible in eight directions: forward, forward-right, right, backward-right, backward, backward-left, left, forward-left. They were instructed to lean as far as possible without losing their balance and return to an upright posture by only pivoting about their ankles only and not bending their knees or hips.

Gait: Walking trials consisted of participants walking along the walkway (9.8 × 1.2 × 0.1 m) at a purposeful pace. Gait speed was semi-controlled by telling participants to speed up or slow down if they deviated by + \− 10% of their preferred walking speed (which was determined before assessments).

Lifting/lowering: Participants completed two trials of a lift, carry, and lower task of a load set at 10% of their body mass with a box placed on floor level.

**Fig. 2:**
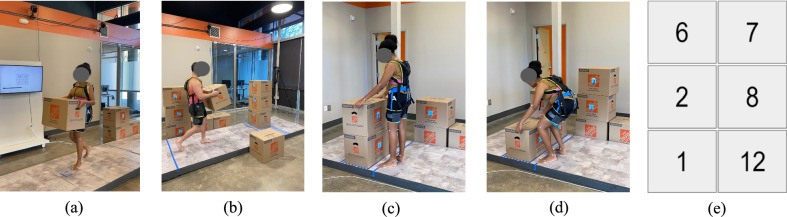
**a**–**d** Illustration of the experimental setup showing lifting, carrying, and walking during the familiarization task as well as **e** an example of the predetermined arrangement of boxes that participants replicated during the familiarization task. Cardboard boxes were each labeled with a number and participants had to arrange the boxes to match the diagram

Familiarization tasks: During each cycle, participants performed industrially relevant manual material handling tasks where they had to rearrange lightweight (0.77 kg) cardboard boxes in a predetermined arrangement on each end of the walkway (Fig. 2). During this activity, participants had to lift, carry, and lower the lightweight boxes at several heights (38, 76, and 114 cm) to complete the predetermined arrangement. We restricted this familiarization period to 15 min and included lightweight boxes (instead of heavy boxes) to expose participants to a variety of manual material handling task configurations while limiting physical fatigue.

### Data Collection and Processing

Whole-body kinematics and ground reaction forces were measured throughout the experiment. Reflective markers (*n* = 33) and one rigid marker cluster (on the sacrum) were sampled at 100 Hz using a 12-camera motion capture system (Qualisys, Inc., Gothenburg, Sweden) then low pass filtered (6 Hz cutoff; 4th-order Butterworth; bidirectional). The custom full-body marker set used in this study is illustrated in Appendix Fig. 7. A force platform (AMTI, Watertown, MA, USA) embedded in the walkway was used to measure triaxial ground reaction forces at 1000 Hz, low pass filtered (6 Hz cutoff; 2nd-order Butterworth; bidirectional), and down-sampled to 100 Hz, and then used to compute the center of pressure (COP) time series [46].

### Data Analysis and Outcome Measures

#### Lifting/Lowering

The thorax segment was defined according to ISB recommendations [47], with a modification where the midpoint between the right and left ASIS markers was used instead of the midpoint between Processus Xiphoideus (PX) and the T8 segment. This modification was made as the exoskeleton made it difficult to reliably track PX and T8 markers across all trials. To calculate trunk angles, we used the standard ZXY rotation sequence of the thorax with respect to vertical, as recommended by ISB [47], with an order of rotation of flexion-extension, lateral bending, and axial rotation. Repetitive lifting/lowering cycles were segmented based on a velocity thresholding algorithm [48], for computation of means and cycle-to-cycle standard deviations of peak trunk flexion angle and velocity. Outcome measures from lifting/lowering tasks included peak trunk flexion angle, trunk range of motion, peak trunk flexion velocity, and the time to reach peak trunk flexion angle (normalized to task duration), which were computed by averaging each measure across the ~ 6 lifting and lowering cycles per participant per assessment. In addition, the cycle-to-cycle standard deviations (SD) of each measure were extracted across the six repeats of lifting/lowering cycles, as increased cycle-to-cycle variability in lifting/lowering kinematics would be indicative of less stable movement patterns during exoskeleton-use compared to no-exoskeleton conditions.

#### Gait

Approximately 16-20 gait cycles were used for computing gait outcome measures at each assessment time point for each participant. Gait events (i.e., heel-strike and toe-off of each foot) were detected using an acceleration-based algorithm applied to the heel (calcaneus) markers [49]. From each gait cycle, we computed the following spatiotemporal measures: step length, step width, swing time, stance time, and minimum toe clearance (MTC) [50]. Step width was calculated as the horizontal distance between heel markers in the transverse plane; step length was calculated as the vertical distance between heel markers in the transverse plane (normalized to the participant’s height); stride length was calculated as the horizontal distance between the heel marker between two heel strikes; and swing time was calculated as the time from toe-off to heel strike whereas stance time was calculated as the time from heel strike to toe-off. MTC was defined as the shortest vertical distance between the ground and the lowest point under the front of the foot (toe marker), occurring during the mid-swing phase of the gait cycle [51, 52]. A decrease in MTC is indicative of increased risk of trips and falls [53, 54].

Kinematics of the bilateral foot, shank and thigh segments were calculated based on ISB recommendations [47]. Three-dimensional rotations of each segment were defined using the ZXY convention relative to the laboratory reference frame, joint angles were calculated as Euler angles between adjacent local segments with an order of rotation of flexion-extension, abduction-adduction, and internal-external rotation. Hip joint angles were computed with respect to the global reference frame. Sagittal plane range of motion and peak angular velocities of the hip, knee, and ankle were computed as outcome measures. All outcome measures were averaged across the multiple gait cycles of data for each participant, for each assessment.

#### Postural Stability

The COP time series from each standing balance trial was used to compute COP mean velocity (MV) and the root mean square distance (RMSD), according to the procedures described in Prieto et al. [55]. Maximum leaning trials were used for estimating the functional limit of stability (FLoS): FLoS was determined by identifying the COP location furthest from the center in the anterior-posterior (AP) and medial-lateral (ML) directions to define a quadrilateral. The area of this quadrilateral was then computed and expressed as a percentage of the base of support (BOS) area in bipedal standing [56]. A larger FLoS area indicates greater functional limits of stability [56, 57].

#### Subjective Perception of Exoskeleton Use

The RPE scale, ranging from 6 to 20, captures the level of exertion participants feel during physical tasks, with 6 representing “no exertion at all” and 20 indicating “maximum exertion.” Similarly, the RPD, ranging from 0 to 10, captures the level of discomfort participants feel at specific body locations, with 0 signified “no discomfort” and 10 reflects “maximum discomfort.” The RPS scale, ranging from 1 to 10, considers how stable a participant feels related to their balance, with 1 denoting feeling “completely stable,” while 10 indicates feeling “about to fall.” Lastly, for evaluating technology acceptance, we applied the Technology Acceptance Model (TAM) questionnaire [58], which includes a 5-point scale ranging from “extremely likely” to “extremely unlikely”

### Statistical Analysis and Modeling

**Fig. 3:**
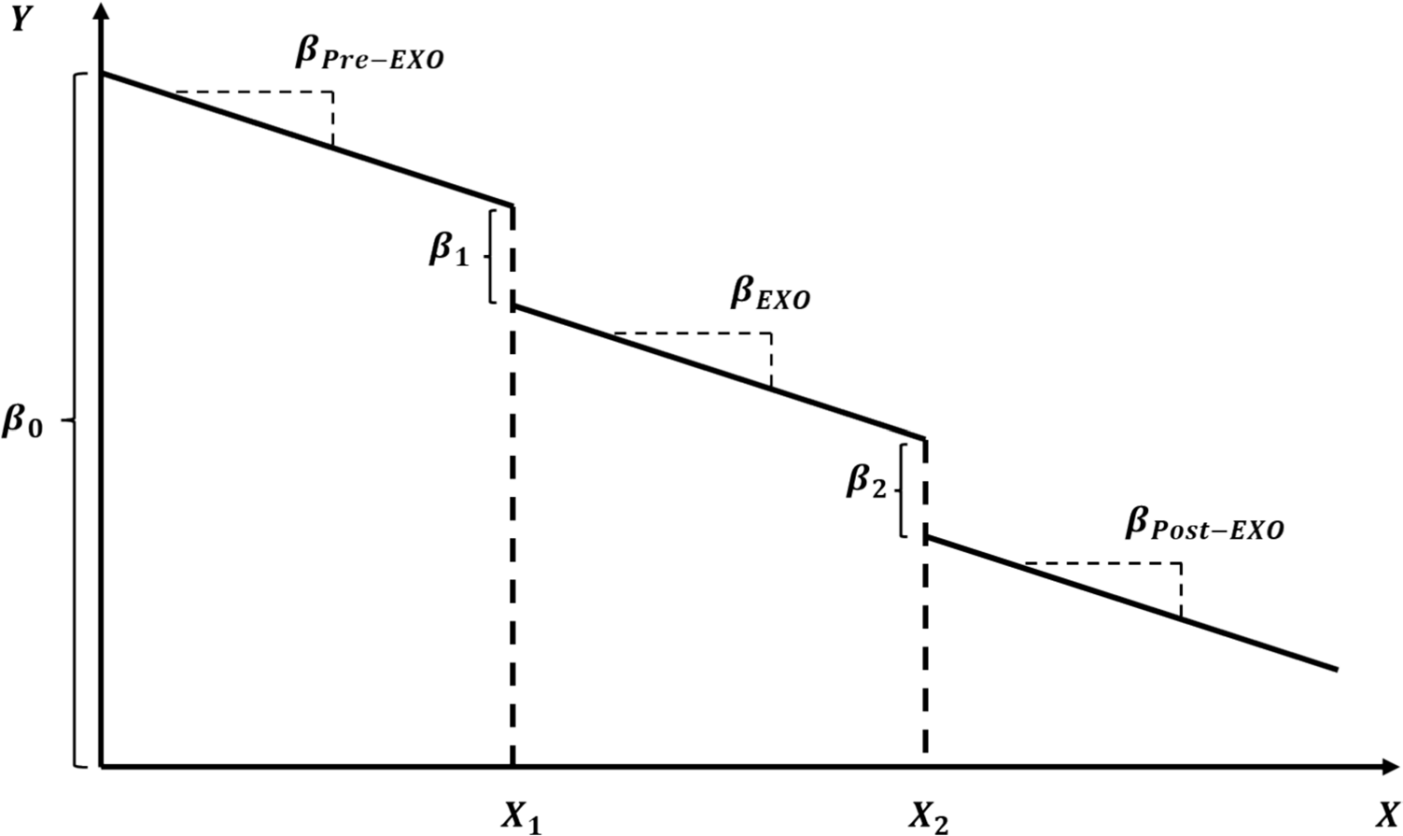
Illustration of the piecewise regression statistical model

The effect of exposure time on the objective outcomes was modeled using a piecewise linear regression model—the data were split into three distinct segments, with each segment representing a different exposure category (*Pre-EXO/EXO-adaptation/Post-EXO*) and hence fit with a separate line. This approach was selected as it allows for analysis of different trends in the outcomes (adaptation measures), such that the effects of exposure time change at specific “breakpoints” within the data range representing the changes in exposure (donning and doffing of exoskeleton). The model was formulated as follows:

$${Y}_{i}={\beta }_{0}+{\beta }_{i1}*{t}_{i}+{\beta }_{1}*{X}_{1}+{\beta }_{i2}*\left({t}_{i}-45\right)*{X}_{1}+{\beta }_{2}{*X}_{2}+{\beta }_{i3}*\left({t}_{i}-120\right)*{X}_{2}+ {\varepsilon }_{i}$$

$${\beta }_{pre-EXO}={ \beta }_{i1}$$

$${\beta }_{EXO}= {\beta }_{i1}+{\beta }_{i2}$$

$${\beta }_{post-EXO}={\beta }_{i1}+{\beta }_{i2}+{\beta }_{i3}$$

where ${y}_{i}$ is an assessment outcome at time instant ${t}_{i}$, ${\beta }_{0}$ is the intercept, ${\beta }_{pre-EXO}$ is the slope of the linear fit to data from the *Pre-EXO* session, ${\beta }_{1}$ represents the change in intercept due to the “breakpoint” representing the introduction of the exoskeleton, ${\beta }_{EXO}$ represents the slope of the linear fit to data from the *EXO-adaptation* session, X_1_ is a qualitative indicator variable (coded as 0 if ${t}_{i}$ ≤ 45; 1 otherwise), ${\beta }_{2}$ represents the change in intercept due to the “breakpoint” representing the removal (i.e., doffing) of the exoskeleton, ${\beta }_{post-EXO}$ represents the slope of the linear fit to data from the *Post-EXO* session, X_2_ is a qualitative indicator variable (coded as 0 if ${t}_{i}$ ≤ 120; 1 otherwise), and ${\varepsilon }_{i}$ is random error. Note that this function is designed to reflect a slope change at approximately 45 min (donning exoskeleton), and at approximately 120 min (doffing exoskeleton) (Fig. 3).

Whether the donning or doffing of the exoskeleton significantly changed an outcome measure was assessed by testing whether ${\beta }_{1}$ or ${\beta }_{2}$ were significantly different from zero (respectively). A non-zero slope during the exoskeleton adaptation phase (i.e., whether ${\beta }_{EXO}$ was significantly different from zero) was used to test for a significant exoskeleton-adaptation effect on the outcome measure of interest; while a non-zero slope in the pre-EXO and post-EXO adaptation phases indicated learning effects in the pre-exoskeleton adaptation session and recovery from recent exoskeleton exposure respectively. Estimates of all intercepts and slopes, their standard errors, and hypothesis tests were performed using JMP, version 18 (SAS Institute Inc., Cary, NC). Separate repeated-measures analyses of variance (ANOVAs) were used to assess the effects of Intervention on the subjective outcome measures (i.e., RPDs, RPE, and RPS).

## Results

**Fig. 4:**
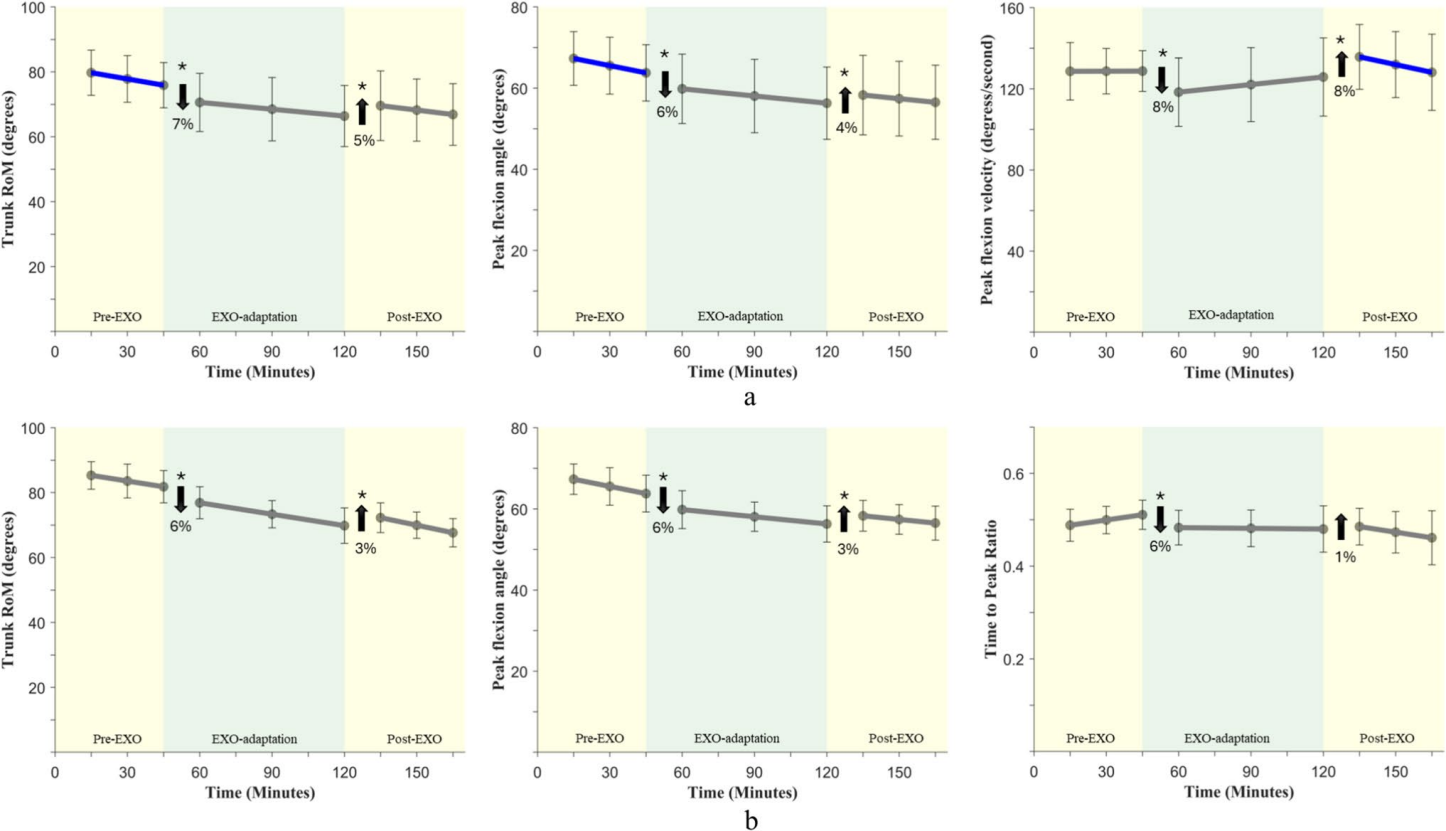
From left to right: Trunk range of motion, peak trunk flexion angle, and peak flexion angular velocity are shown in the top and bottom panels. **a** Trunk kinematics during lifting and **b** Trunk kinematics during lowering. Data are presented as least-squares means with error bars indicating 95% confidence intervals. The star symbol (*) indicates statistical significance, and the blue highlights on lines indicate a significant change in the slope

**Fig. 5:**
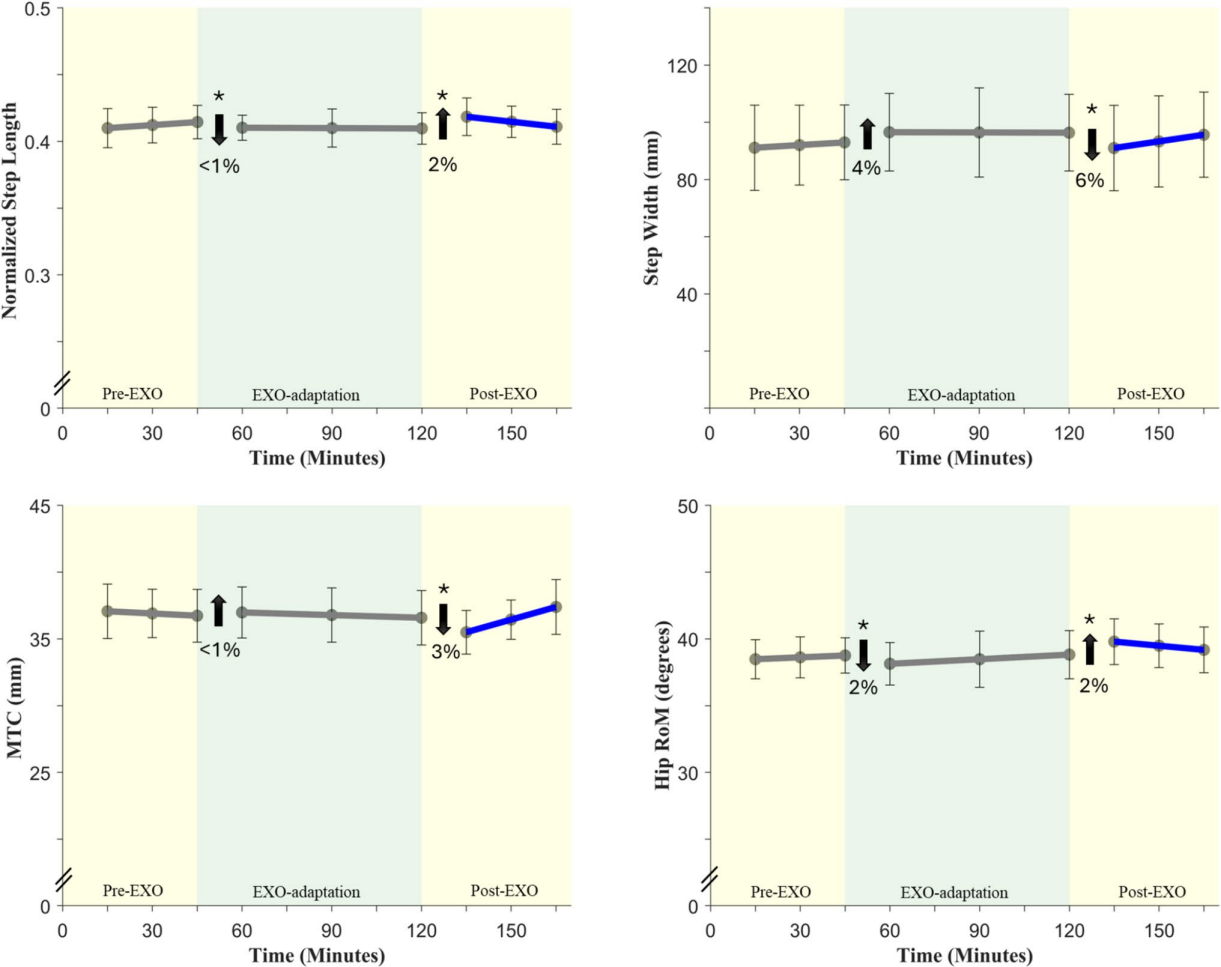
Spatiotemporal gait parameters. Data are presented as least-squares means with error bars indicating 95% confidence intervals. The star symbol (*) indicates statistical significance, and the blue line indicates a significant change in the slope

**Fig. 6:**
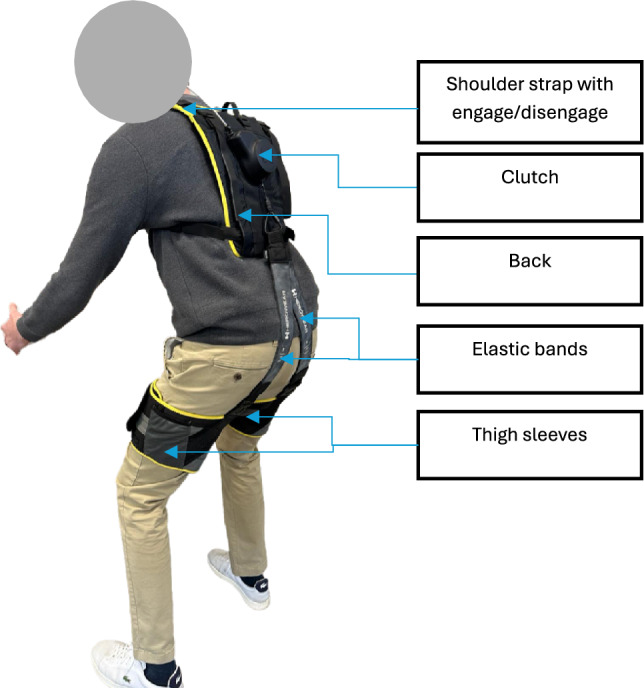
HeroWear Apex passive back support exoskeleton with key components marked and labeled

The piece-wise linear regression results are presented by main outcome categories: lifting/lowering (Table 2; Fig. 4), gait (Table 3; Fig. 5), and postural stability (Appendix Table 6).

**Table 2:** Summary of piece-wise regression results for all the slopes and intercepts on each of the trunk outcome measures during lifting and lowering

Outcome measures		${\beta }_{Pre-EXO}$	${\beta }_{1}$	${\beta }_{EXO}$	${\beta }_{2}$	${\beta }_{Post-EXO}$
*Lifting*						
RoM	*p*-value	**0.02**	** < 0.001**	0.35	** < 0.001**	0.75
	Std. β	**− 0.32**	**− 0.18**	− 0.19	**0.20**	− 0.21
Peak flexion angle	*p*-value	**0.02**	** < 0.001**	0.29	**0.002**	0.99
	Std. β	**− 0.31**	**− 0.13**	− 0.17	**0.14**	− 0.18
Peak flexion velocity	*p*-value	0.98	** < 0.001**	0.47	** < 0.001**	0.02
	Std. β	0.01	**− 0.30**	0.16	**0.26**	− 0.01
Time to peak ratio	*p*-value	0.39	0.15	0.07	0.74	0.16
	Std. β	0.34	− 0.09	0.16	0.10	0.06
SD_RoM	*p*-value	0.60	− 0.27	0.26	0.35	0.52
	Std. β	0.34	0.21	− 0.38	0.21	− 0.24
SD_Peak flexion angle	*p*-value	0.21	0.26	0.49	0.29	0.30
	Std. β	0.57	0.28	− 0.64	0.08	− 0.32
SD_Peak flexion velocity	*p*-value	0.09	0.44	0.17	0.53	0.38
	Std. β	− 1.17	0.15	− 0.25	0.15	− 0.04
SD_Time to peak ratio	*p*-value	0.95	0.31	0.67	0.45	0.74
	Std. β	− 0.03	0.14	0.16	− 0.12	0.11
*Lowering*						
RoM	*p*-value	0.06	**0.001**	0.99	** < 0.001**	0.61
	Std. β	− 0.29	**− 0.15**	− 0.29	**0.20**	− 0.27
Peak flexion angle	*p*-value	0.14	**0.001**	0.75	** < 0.001**	0.67
	Std. β	− 0.21	**− 0.13**	− 0.26	**0.17**	− 0.24
Peak flexion velocity	*p*-value	0.92	0.46	0.87	0.74	0.77
	Std. β	0.02	0.05	− 0.02	0.03	0.01
Time to peak ratio	*p*-value	0.16	** < 0.001**	0.17	0.34	0.23
	Std. β	0.36	**− 0.28**	0.01	0.08	0.12
SD_RoM	*p*-value	0.41	0.05	0.56	0.98	0.45
	Std. β	− 0.48	0.33	− 0.15	0.01	− 0.30
SD_Peak flexion angle	*p*-value	0.63	0.05	0.92	0.89	0.95
	Std. β	− 0.30	0.36	− 0.24	0.03	− 0.25
SD_Peak flexion velocity	*p*-value	0.83	0.85	0.72	0.60	0.19
	Std. β	0.15	0.04	0.40	− 0.13	0.08
SD_Time to peak ratio	*p*-value	0.78	0.37	0.45	0.11	0.62
	Std. β	− 0.15	0.14	0.25	− 0.30	0.34

In each cell, *p* values are reported along with effect sizes (*standard beta* values); significant effects are highlighted in bold (*p* < 0.05)

**Table 3:** Summary of piece-wise regression results for slopes and intercepts on each outcome measure during gait

Outcome Measures		${\beta }_{Pre-EXO}$	${\beta }_{1}$	${\beta }_{EXO}$	${\beta }_{2}$	${\beta }_{Post-EXO}$
*Spatiotemporal gait measures*						
Step Length	*p*-value	0.17	**0.006**	0.20	** < 0.001**	**0.05**
	Std. β	0.25	**− 0.15**	0.02	**0.30**	**− 0.10**
Step Width	*p*-value	0.26	0.08	0.19	** < 0.001**	**0.05**
	Std. β	0.18	0.08	− 0.02	**− 0.15**	**0.08**
Stance Time	*p*-value	0.37	0.37	0.08	0.93	0.05
	Std. β	0.32	0.09	− 0.28	− 0.01	− 0.06
Swing Time	*p*-value	0.72	0.14	0.20	0.89	0.06
	Std. β	0.13	− 0.15	0.59	− 0.02	0.36
*Trip risk*						
MTC	*p*-value	0.66	0.36	0.87	**0.005**	**0.01**
	Std. β	− 0.13	0.08	− 0.08	**− 0.28**	**0.16**
*RoM*						
Hip	*p*-value	0.49	** < 0.001**	0.88	** < 0.001**	**0.03**
	Std. β	0.13	**− 0.19**	0.15	**0.23**	**0.02**
Knee	*p*-value	0.14	0.08	0.25	0.33	0.65
	Std. β	0.31	− 0.11	0.07	0.07	0.04
Ankle	*p*-value	0.47	0.24	0.65	0.15	0.84
	Std. β	0.06	− 0.03	0.02	0.04	0.03
*Peak angular velocity*						
Hip extension	*p*-value	0.68	0.96	0.57	0.56	0.07
	Std. β	0.24	− 0.01	− 0.06	0.10	0.25
Hip flexion	*p*-value	0.65	0.70	0.77	0.89	0.59
	Std. β	0.26	− 0.06	0.10	0.03	0.01
Knee extension	*p*-value	0.17	0.87	0.17	0.88	0.38
	Std. β	0.67	− 0.02	− 0.01	− 0.03	0.15
Knee flexion	*p*-value	0.98	0.69	0.88	0.64	0.69
	Std. β	− 0.05	0.07	0.04	0.08	0.12
Ankle plantarflexion	*p*-value	0.75	0.31	0.45	0.06	0.58
	Std. β	0.14	0.12	− 0.17	− 0.27	− 0.25
Ankle dorsiflexion	*p*-value	0.81	0.68	0.98	0.58	1.00
	Std. β	0.09	-0.04	0.08	0.07	0.08

In each cell, *p* values are reported along with effect sizes (*standard beta* values); significant effects are highlighted in bold (*p* < 0.05). *Norm.* normalized, *GC* gait cycle

### Lifting/Lowering

Several trunk kinematic parameters showed significant changes when donning the exoskeleton (i.e., during transition from *Pre-EXO* to *EXO*), and also immediately after doffing the exoskeleton (i.e., when transitioning from *EXO-adaptation* to *Post-EXO*). However, none of these measures showed significant adaptation effects during the *EXO-adaptation* phase. The descriptive summaries of all measures are reported in Appendix Table 4, the changes with donning the exoskeleton and use are illustrated in Fig. 4, and results from the statistical tests are reported in Table 2. During lifting (top panel, Fig. 4a), in addition to small changes in trunk kinematics during the *Pre-EXO* phase, trunk RoM, peak trunk flexion angle, and peak trunk flexion velocity decreased significantly, by 6-8%, when donning the EXO (*Pre-EXO* to *EXO-adaptation* transition) as compared to *Pre-EXO* baseline values. Furthermore, trunk RoM, peak trunk flexion angle and trunk flexion velocity also increased significantly (towards baseline levels, by 4-8%), when doffing the exoskeleton; however, their recovery during the *Post-EXO* phase was not significant (non-significant *Post-EXO* slope, Table 2). Similar changes were also observed during the lowering task (bottom panel, Fig. 4b), with the exception that time-to-peak ratio was also significantly affected when donning the exoskeleton (compared to *Pre-EXO*). The cycle-to-cycle standard deviations of trunk RoM, peak trunk flexion angle and velocity, and time to peak ratio decreased (by ~ 6%) on donning the exoskeleton and increased (by ~ 1%) on doffing the exoskeleton; however, these changes were not statistically significant.

### Gait Parameters

Several gait parameters showed significant changes on donning the exoskeleton (i.e., during transition from *Pre-EXO* to *EXO*), immediately after doffing the exoskeleton (i.e., when transitioning from *EXO-adaptation* to *Post-EXO*), and some carry-over effects (i.e. *Post-EXO* slope) also persisted after doffing the exoskeleton. However, none of these measures showed significant adaptation effects during the exoskeleton use-time (i.e. *EXO-adaptation* slope). The descriptive summaries of all measures are reported in Appendix Table 5. Changes when donning the exoskeleton introduction and use are illustrated in Fig. 5, and results from the statistical tests are reported in Table 3. During gait, step length and hip RoM decreased significantly, by 1–2%, on donning the exoskeleton (as compared to *Pre-EXO* baseline values) and increased by 2–3% on doffing the exoskeleton. Step width and MTC decreased by 6 and 3% respectively, on doffing the exoskeleton. All four measures showed significant changes in the *Post-EXO* phase, indicating a carry-over effect of having worn the exoskeleton for a prolonged duration.

### Postural Stability

Exoskeleton use had significant effects on postural stability during unipedal stance (Table 6). COP mean velocity in the AP direction (MV_AP_) decreased over time during the *Pre-EXO* condition and decreased during *EXO-adaptation* condition. Other measures such as COP mean velocity in the ML direction (MV_ML_) and FLoS_AREA_ only showed changes in the *Pre-EXO* condition and *Post-EXO* conditions. The descriptive summaries of all measures are reported in Appendix Table 7.

### Subjective Measures

No statistically significant differences were observed in RPE, RPD, and RPS measures across conditions (Table 8). No significant differences were observed in the modified TAM questionnaire that included Perceived Usefulness (PU) and Perceived Ease of Use (PEU) as well (Tables 9, 10).

## Discussion

To the best of our knowledge, this is the first study that has aimed to characterize movement kinematic adaptations during lifting, gait, and postural control to a passive back-support exoskeleton over 75 min of exposure. We hypothesized that when initially introduced to the exoskeleton, users may alter lifting/lowering strategies, gait, and balance, due to any device-induced movement restrictions and external assistance. Over the 75-min exoskeleton exposure period, we expected individuals to gradually adapt their movement kinematics with increased exposure. This was tested using multiple assessments over the time-course of the exposure. Upon removing the exoskeleton, we expected to see some carry-over effects in lifting/lowering strategies, gait, and balance before gradually returning to baseline movement patterns, during the post-exoskeleton-use period. These carry-over effects were expected to reflect a transition from any new movement kinematics that users had adopted (to perform tasks when using the exoskeleton), which may dissipate or persist, depending on the extent of adaptation.

### Lifting/Lowering

During the lifting/lowering tasks, introduction of the exoskeleton led to significant reductions in trunk RoM (~ 7%), peak trunk flexion angle (~ 6%), and peak trunk flexion velocity (~ 8%), compared to the no-exoskeleton baseline. Similar results have been reported in the past for the Apex exoskeleton—Goršič et al. [23] and Raghuraman et al. [20] reported a 5–10% reduction in trunk RoM, Raghuraman et al. [20] reported a 6% reduction in peak trunk flexion angle and 8% reduction in peak trunk velocity [20]. When considering doffing effects, there are very few studies that have explored the immediate effects of removing an exoskeleton’s assistance by disengaging or removing an exoskeleton. Goršič et al. [23] observed a 3–13% increase in trunk RoM after disengaging exoskeleton assistance, which is somewhat similar to our finding of a 5% increase after doffing the exoskeleton. We also observed a 4% increase in peak flexion angle and 8% increase in peak flexion velocity after doffing the exoskeleton, but there were no available data for comparisons. Most notably, none of the trunk kinematic measures during lifting or lowering exhibited any significant adaptation effects over the time course of the exposure. Cycle-to-cycle variability of trunk flexion angle and velocity, and time to peak ratio also did not change significantly over the course of the experiment.

While prior work has shown similar small (< 10–15%) changes in trunk kinematics with exoskeleton-use, whether these small magnitudes of changes truly reflect meaningful alterations to movement strategies, and/or if they will simply dissipate over time as users get accustomed to the device, have been questioned [8, 26]. Even if small in magnitude, our findings that trunk ROM, peak flexion angle, and velocity decreased on donning the exoskeleton and significantly reversed (i.e., increased) immediately on doffing the exoskeleton, indicate that there are clear alterations (restrictions) to trunk kinematics when using the exoskeleton. Furthermore, since none of these measures exhibited significant adaptation during exoskeleton use, and since movement variability was also unchanged during the adaptation period, these altered trunk kinematics may be stable and not diminish over time, at least within a single session lasting ~ 1 h.

### Gait Parameters

Similar to lifting/lowering kinematics, there were significant changes in specific gait measures on donning the exoskeleton and significant reversal of the same changes on doffing the exoskeleton. In general, people seem to have exhibited a more cautious gait pattern with exoskeleton-use, since step length decreased, step width increased, MTC increased and hip ROM decreased with exoskeleton introduction. The cautious gait patterns observed during exoskeleton use may be attributed to the external hip extension torque created by the device and are in accordance with prior findings. Park et al. [29] and Baltrusch et al. [15] reported comparable magnitudes of reduction in step length, stride length, and hip ROM when testing different exoskeleton designs. Similar to trunk kinematics during lifting, none of the gait measures showed significant adaptation over the exoskeleton exposure period. However, most interestingly, when the exoskeleton was removed, people seem to have “over-compensated” their gait in returning to baseline. This was evident from the gait measures shown in Fig. 5: For example, step length increased significantly on doffing the exoskeleton, followed by a significant carry-over effect during the *Post-EXO* phase (significant Post-EXO slope), to return to baseline (*Pre-EXO* levels). Step width, MTC and hip ROM showed similarly significant carry-over effects after doffing the exoskeleton.

### Postural Stability

Among the various postural stability measures quantified from unipedal stance and maximal leans, some small changes were noted in the COP mean velocity in the AP and ML directions (MV_AP_ and MV_ML_) during the *Pre-EXO* phase, COP MV_AP_ during exoskeleton adaptation, and in the functional limit of stability during the *Post-EXO* phase. However, our overall findings largely indicated that exoskeleton introduction, prolonged use, and doffing did not alter postural stability in any significant manner.

*Subjective perceptions* of exoskeleton use consisting of RPE, RPD, RPS, and TAM scores did not change significantly over time.

### Limitations

This study was conducted with a convenience sample of twelve healthy young adults, and to what extent our results can be generalized to broader populations, such as older adults, is currently not known. Second, the controlled laboratory environment in which the study was conducted does not replicate the complexities of real-world environments. Tasks performed in such controlled settings may not reflect the natural variability and unpredictability of actual occupational tasks, which can significantly affect the perception and effectiveness of exoskeletons in practice.

### Conclusion

In summary, while our findings of decreased trunk flexion and ROM restrictions and a more cautious gait pattern with exoskeleton introduction agreed with prior findings in the literature, we did not find any significant adaptations in any of the measures tested in this study over the 75 min of exposure. We also discovered carry-over effects in gait on doffing the exoskeleton. It is not clear from our data whether any longer-term changes to movement behaviors may have occurred over exposure durations of the order of several hours or over multiple days/sessions occurring over several weeks/months. In current industrial practice, many exoskeleton manufacturers currently recommend a graded familiarization protocol, starting with small periods of exposure to the exoskeleton and ramping up the exposure duration over a multi-week period. We are not aware of the scientific basis for this protocol and to what extent it is effective, with the exception of the recent study by Jakobsen and colleagues [33]. Future work, including objective and subjective outcomes of exoskeleton use, spanning over multiple sessions, is required to confirm whether longer-term motor learning occurs when using passive back-support exoskeletons for occupational use.

## Figures and Tables

**Fig. 7:**
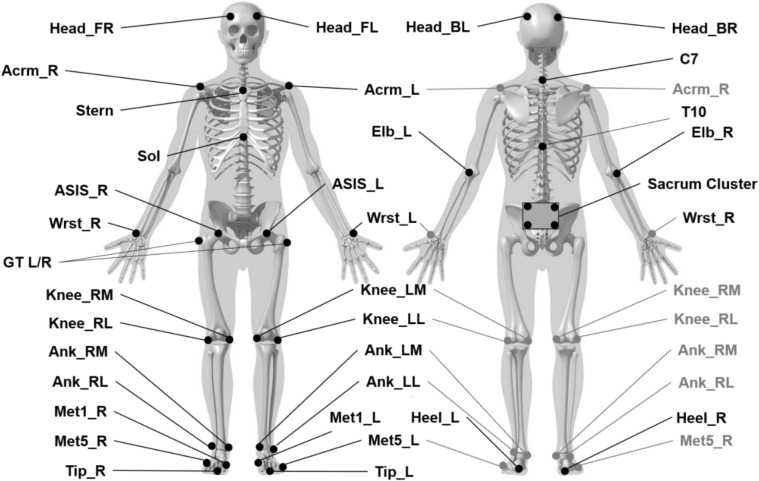
Anterior (left) and posterior (right) views of the marker set for kinematics analysis. Tip = 1st distal phalanx; (Heel) = calcaneus; Met1 = 1sr metatarsal head; Met5 = 5th metatarsal head; Ank = malleolus; Knee = femoral epicondyle; GT = greater trochanter; ASIS = anterior superior iliac spine; Stern = suprasternale; Sol = xiphoid process; C7 = spinous process of the 7th cervical vertebra; Acrm = acromium; Elb = olecranon; Wrst = radial styloid; Sacrum Cluster. Participants wore a hairband where four reflective markers (i.e., Head_FR, Head_FL, Head_BL, Head_BR) were attached

## Appendix

See Figs. 6, 7 and Tables 4, 5, 6, 7, 8, 9, 10.

**Table 4:** Descriptive summaries of all measures

Measure	Pre-EXO: mean (SD)	EXO-adaptation: mean (SD)	Post EXO: mean (SD)
*Lifting*			
RoM (°)	**77.69 (1.93)**	65.02 (2.14)	67.99 (1.25)
Peak flexion angle (°)	**65.65 (1.64)**	56.01 (1.78)	57.38 (0.87)
Peak flexion velocity (°s^−1^)	130.76 (0.87)	114.35 (3.93)	128.32 (3.97)
Time to peak ratio (%)	0.48 (0.01)	0.48 (0.01)	**0.50 (0.01)**
SD_RoM (°)	6.20 (0.66)	6.62 (0.86)	4.35 (0.21)
SD_Peak flexion angle (°)	5.11 (0.55)	5.33 (1.34)	3.95 (0.31)
SD_Peak flexion velocity (°s^−1^)	18.15 (3.81)	16.81 (1.93)	16.10 (1.76)
SD_Time to peak ratio (%)	0.049 (0.002)	0.071 (0.006)	0.065 (0.003)
*Lowering*			
RoM (°)	82.42 (1.83)	68.98 (3.27)	70.50 (2.02)
Peak flexion angle (°)	69.65 (1.15)	58.21 (2.66)	59.12 (1.86)
Peak flexion velocity (°s^−1^)	108.43 (3.89)	111.38 (1.04)	111.98 (0.92)
Time to peak ratio (%)	0.52 (0.01)	0.47 (0.01)	0.50 (0.01)
SD_RoM (°)	5.19 (0.83)	7.14 (1.05)	5.71 (0.91)
SD_Peak flexion angle (°)	4.37 (0.53)	6.16 (0.87)	4.95 (0.58)
SD_Peak flexion velocity (°s^−1^)	12.99 (0.61)	19.81 (5.25)	15.02 (1.87)
SD_Time to peak ratio (%)	0.06 (0.01)	0.08 (0.01)	0.08 (0.01)

Values that were found to be significant (*p* < 0.05) are highlighted in bold font

**Table 5:** The descriptive summaries of all measures

Measure	Pre-EXO: mean (SD)	EXO-adaptation: mean (SD)	Post EXO: mean (SD)
*Spatiotemporal gait measures*			
Step Length (Norm.)	0.41 (0.01)	0.40 (0.01)	0.41 (0.01)
Step Width (mm)	95.42 (1.93)	101.76 (2.25)	95.45 (3.45)
Stance Time (%GC)	0.59 (0.01)	0.60 (0.01)	0.60 (0.01)
Swing Time (%GC)	0.41 (0.01)	0.40 (0.010)	0.40 (0.01)
*Trip risk*			
MTC (mm)	37.88 (0.70)	38.14 (0.69)	37.17 (1.00)
*RoM*			
Hip (°)	38.54 (0.05)	37.57 (0.36)	39.20 (0.37)
Knee (°)	61.27 (0.21)	60.69 (0.04)	60.75 (0.48)
Ankle (°)	46.08 (3.80)	40.59 (2.09)	42.13 (1.91)
*Peak angular velocity*			
Hip extension (°s^−1^)	99.47 (7.78)	99.69 (10.68)	96.58 (7.67)
Hip flexion (°s^−1^)	272.98 (28.94)	258.32 (21.38)	267.06 (47.07)
Knee extension (°s^−1^)	231.27 (21.53)	221.65 (32.10)	225.02 (32.32)
Knee flexion (°s^−1^)	197.66 (60.63)	147.31 (16.33)	167.96 (4.97)
Ankle plantarflexion (°s^−1^)	417.00 (66.62)	386.97 (14.90)	401.40 (75.35)
Ankle dorsiflexion (°s^−1^)	226.48 (34.27)	224.59 (38.22)	242.41 (46.66)

**Table 6:** Summary of piecewise regression results for all the slopes and intercepts on each of the outcome measures during unipedal stance and maximum lean

Outcome measures		${\beta }_{Pre-EXO}$	${\beta }_{1}$	${\beta }_{EXO}$	${\beta }_{2}$	${\beta }_{Post-EXO}$
*Unipedal stance*						
MV_AP_	*p*-value	**0.001**	0.42	**0.02**	0.61	0.43
	Std. β	**− 1.12**	0.08	**− 0.22**	− 0.06	− 0.22
MV_ML_	*p*-value	**0.005**	0.16	0.07	0.64	0.53
	Std. β	**− 1.07**	− 0.16	− 0.06	0.06	0.02
RMSD_AP_	*p*-value	0.45	0.33	0.66	0.25	0.73
	Std. β	− 0.37	0.14	− 0.16	0.19	− 0.21
RMSD_ML_	*p*-value	0.53	0.63	0.51	0.90	0.98
	Std. β	− 0.32	− 0.07	0.01	− 0.02	0.01
*Maximum lean*						
FLoS_AREA_	*p*-value	0.18	0.81	0.05	0.21	**0.04**
	Std. β	− 0.05	− 0.03	0.71	− 0.16	**− 0.27**

In each cell, *p* values are reported along with standard beta, with bold font highlighting significant effects (*p* < 0.05)

**Table 7:** The descriptive summaries of all measures

Measure	Pre-EXO: Mean (SD)	EXO-Adaptation: Mean (SD)	Post EXO: Mean (SD)
*Unipedal*			
MV_AP_	2.23 (0.17)	2.09 (0.05)	2.12 (0.03)
MV_ML_	2.67 (0.20)	2.36 (0.04)	2.56 (0.07)
RMSD_AP_	0.83 (0.02)	0.85 (0.02)	0.88 (0.05)
RMSD_ML_	0.72 (0.12)	0.64 (0.04)	0.65 (0.01)
*Maximum lean*			
MV_AP_	3.18 (0.15)	3.28 (0.04)	3.58 (0.01)
MV_ML_	2.59 (0.09)	2.65 (0.09)	2.94 (0.04)
RMSD_AP_	3.43 (0.15)	3.64 (0.07)	3.81 (0.02)
RMSD_ML_	2.58 (0.03)	2.73 (0.04)	2.90 (0.03)
FLoS_AREA_	0.43 (0.02)	0.45 (0.02)	0.47 (0.02)

**Table 8:** Summary of ANOVAs for subjective measures (RPE, RPD and RPS)

	Pre-EXO to EXO-adaptation	During EXO-adaptation	EXO-adaptation to Post-EXO
*RPE*			
Mean	9.83 to 10.71	10.71 to 10.88	10.88 to 10.46
Std Error	0.8092	0.8092	0.8092
Tukey HSD *p*-value	0.67	0.99	0.98
*RPD—Shoulder*			
Mean	0 to 0.83	0.83 to 1.58	1.58 to 0.58
Std Error	0.3178	0.3178	0.3178
Tukey HSD *p*-value	0.35	0.47	0.17
*RPD—Upper back*			
Mean	0.67 to 0.75	0.75 to 0.41	0.41 to 0.58
Std Error	0.2661	0.2661	0.2661
Tukey HSD *p*-value	0.99	0.80	0.99
*RPD—Lower back*			
Mean	1.25 to 1.33	1.33 to 1.08	1.08 to 1.08
Std Error	0.5294	0.5294	0.5294
Tukey HSD *p*-value	1.00	0.99	1.00
*RPD—Legs*			
Mean	1.25 to 1.00	1.00 to 1.75	1.75 to 1.50
Std Error	0.48904	0.48904	0.48904
Tukey HSD *p*-value	0.99	0.71	0.99
*RPS*			
Mean	2.17 to 2.42	2.42 to 2.25	2.25 to 2.42
Std Error	0.41981	0. 41981	0. 41981
Tukey HSD *p*-value	0.99	0.99	0.99

**Table 9:** Summary of ANOVA for technology acceptance: perceived usefulness

	Using this exoskeleton in my job would enable me to accomplish tasks more quickly	Using this exoskeleton would improve my job performance	Using this exoskeleton would increase my productivity	Using this exoskeleton would enhance my effectiveness on the job	Using this exoskeleton would make it easier to do my job	I would find this exoskeleton useful in my job
Mean ± SD score after F1	3.25 ± 0.87	3.00 ± 1.21	3.00 ± 0.85	3.00 ± 0.74	2.83 ± 1.34	2.92 ± 1.08
Mean ± SD score after F2	2.75 ± 1.06	2.42 ± 1.08	2.58 ± 1.08	2.5 ± 1.00	2.5 ± 1.09	2.58 ± 0.90
Mean ± SD score after F3	2.58 ± 0.90	2.29 ± 1.01	2.33 ± 0.98	2.67 ± 0.98	2.42 ± 0.99	2.42 ± 1.08
*p*-value	0.213	0.258	0.256	0.406	0.647	0.486

**Table 10:** Summary of ANOVA for Technology acceptance: Perceived ease-of-use

	Learning to use this exoskeleton would be easy for me	I would find it easy to get this exoskeleton to do what I want it to do	My interaction with this exoskeleton would be clear and understandable	I would find this exoskeleton to be clear and understandable to use	It would be easy for me to be skillful at using this exoskeleton	[I would find this exoskeleton easy to use
Mean ± SD score after F1	1.75 ± 0.86	2.33 ± 0.89	2.08 ± 1.08	2.00 ± 1.04	1.92 ± 0.90	2.25 ± 1.22
Mean ± SD score after F2	1.83 ± 0.94	2.50 ± 1.00	1.96 ± 1.01	2.00 ± 1.04	2.17 ± 1.03	2.00 ± 1.04
Mean ± SD score after F3	1.58 ± 0.79	1.92 ± 0.99	1.92 ± 1.08	1.91 ± 1.08	2.00 ± 1.13	1.83 ± 0.94
*p*-value	0.774	0.323	0.923	0.975	0.832	0.636
